# Outcomes of Platelet-Rich Plasma Versus Dextrose 10% Prolotherapy in the Treatment of Osgood-Schlatter Disease: A Retrospective Study

**DOI:** 10.7759/cureus.92414

**Published:** 2025-09-15

**Authors:** Rahul Thapa, Arun H Shanthappa, Sunil Chandrashekar, Ayush Agrawal

**Affiliations:** 1 Department of Orthopaedics, Sri Devaraj Urs Medical College, Kolar, IND

**Keywords:** adolescent knee pain, dextrose prolotherapy, osgood-schlatter disease, pediatric orthopedics, pediatric sports medicine, platelet-rich plasma, regenerative medicine, regenerative medicine therapies

## Abstract

Background: Osgood-Schlatter disease (OSD) is a common overuse injury affecting adolescents engaged in physical activities. While conservative treatment remains the standard, refractory cases may benefit from regenerative therapies, such as platelet-rich plasma (PRP) and 10% dextrose prolotherapy (DPT). Comparative evidence between these interventions in OSD is limited.

Objectives: To compare the 12-week clinical effectiveness of single PRP injection versus triple DPT injection series in adolescents with refractory OSD, specifically evaluating pain reduction (primary), functional improvement (primary), return-to-sports duration (secondary), and patient satisfaction (secondary).

Methods: This retrospective comparative cohort study included 40 adolescents with radiologically and clinically diagnosed refractory OSD treated between 2022 and 2024. Treatment allocation was non-randomized based on availability and patient preference. Patients received either a single PRP injection (n=20) or a triple DPT injection series (n=20). Primary outcomes were pain reduction (visual analog scale) and functional improvement (Lysholm Knee Score) measured at baseline, four, eight, and 12 weeks. Secondary outcomes included return-to-sports duration and patient satisfaction. Statistical analysis employed hierarchical Bonferroni correction for primary outcomes (adjusted α=0.0125) with secondary outcomes analyzed at α=0.05.

Results: PRP demonstrated greater pain reduction (VAS: 7.9±0.8 to 1.8±0.9 vs DPT: 7.6±1.0 to 3.4±1.1, p<0.001, Cohen's d=1.60, 95% CI: 1.15-2.05) and functional improvement (Lysholm: 50.8±6.2 to 91.5±4.8 vs 52.1±7.0 to 82.4±5.6, p<0.001, Cohen's d=1.71, 95% CI: 1.25-2.17). PRP patients returned to sports faster (6.1±1.2 vs 8.5±1.3 weeks, p<0.001, Cohen's d=1.94) with higher satisfaction rates (90% vs 65%, p=0.039, OR=5.54, 95% CI: 1.01-30.2).

Conclusion: In this small retrospective study, PRP showed better short-term outcomes than DPT for refractory OSD, including greater pain reduction and faster return to sports. Further randomized controlled trials are warranted to confirm these findings.

## Introduction

Osgood-Schlatter disease (OSD) is a common cause of anterior knee pain in physically active adolescents, resulting from repetitive microtrauma to the developing tibial tuberosity during periods of rapid skeletal growth. The condition affects 6-21% of adolescent athletes, with higher rates in high-impact sports requiring jumping and cutting movements, and typically occurs during growth spurts between ages 10 and 15 years [1,2]. OSD commonly presents unilaterally, though bilateral involvement occurs in approximately 20-30% of cases [3]. The developing tibial tuberosity represents a vulnerable secondary ossification center with reduced mechanical strength compared to mature bone, making it susceptible to traction-induced microtrauma during repetitive quadriceps loading [4,5].

Conservative management remains the cornerstone of OSD treatment and includes activity modification, ice application, nonsteroidal anti-inflammatory drugs, quadriceps and hamstring stretching, and physiotherapy focusing on eccentric strengthening exercises [6,7]. Most patients experience symptom resolution within 12-24 months with conservative approaches, coinciding with skeletal maturity [8]. However, approximately 10-15% of patients develop refractory symptoms lasting beyond three months despite adequate conservative treatment, requiring alternative interventions [9,10].

For refractory cases, regenerative therapies offer promising alternatives to traditional treatments. Dextrose 10% prolotherapy (DPT) utilizes hyperosmolar dextrose solutions (typically 10-25%) to induce controlled inflammatory responses at enthesis sites, promoting tissue repair through fibroblast proliferation and collagen synthesis [11,12]. Treatment involves multiple injections (3-6 sessions) spaced 1-2 weeks apart, with reported success rates of 60-80% in adult tendinopathy studies [13]. Platelet-rich plasma (PRP) therapy concentrates autologous platelets containing supraphysiologic levels of growth factors, including platelet-derived growth factor, transforming growth factor-beta, and vascular endothelial growth factor [14,15]. These bioactive molecules facilitate tissue repair through anti-inflammatory and regenerative mechanisms, typically requiring only a single injection [16].

Despite growing interest in regenerative therapies for adolescent musculoskeletal conditions, comparative evidence between PRP and DPT specifically in OSD remains limited, with most literature focusing on adult populations [17,18]. This retrospective study aimed to compare the 12-week clinical effectiveness of a single PRP injection versus a triple DPT injection series in adolescents with refractory OSD.

Study objectives

Primary Research Question

Do adolescents with refractory OSD treated with PRP demonstrate superior 12-week clinical outcomes compared to those treated with DPT?

Primary Objectives

Pain assessment: Compare pain reduction between PRP and DPT using visual analog scale (VAS) scores at four, eight, and 12 weeks post-treatment.

Functional assessment: Evaluate functional improvement differences using Lysholm Knee scores at four, eight, and 12 weeks post-treatment.

Secondary Objectives

Return to activity: Compare the time to return to unrestricted sports participation between treatment groups.

Patient experience: Assess patient satisfaction rates at 12-week follow-up using a validated Likert scale.

Safety profile: Document adverse events and complications in both treatment groups during the 12-week follow-up period.

Exploratory Objectives

Examine correlations between pain reduction and functional improvement across treatment groups.

Investigate potential treatment response modifiers, including age, gender, sport type, and symptom duration.

Study Hypotheses

Primary hypothesis: PRP injection will demonstrate superior pain reduction and functional improvement compared to DPT series, based on concentrated growth factor content and simplified single-injection treatment protocol.

Secondary hypothesis: PRP will enable faster return to sports and higher patient satisfaction due to single-injection convenience and potentially enhanced biological healing response.

Success Criteria (Pre-defined)

Clinically meaningful pain reduction: ≥2-point VAS decrease from baseline (based on pediatric pain literature).

Functional success: ≥15-point Lysholm score improvement (representing transition between functional categories).

Return to sports success: Resumption of pre-injury activity level within 10 weeks without pain symptoms (VAS ≤2).

Patient satisfaction success: Satisfaction score ≥4/5 on validated Likert scale.

Operational Definitions

Refractory OSD: Symptoms persisting >3 months despite adequate conservative management, including NSAIDs, physiotherapy, and activity modification for a minimum of eight weeks.

Return to sports: Resumption of pre-injury competitive or recreational activity level without functional limitations or pain during participation.

Treatment success: Achievement of both primary success criteria (pain reduction ≥2 VAS points AND functional improvement ≥15 Lysholm points).

Short-term follow-up: 12-week post-intervention assessment period, chosen to capture acute treatment response while minimizing loss to follow-up.

Study Scope and Limitations

This retrospective study focuses on short-term clinical effectiveness (12 weeks) and does not assess long-term outcomes, cost-effectiveness, optimal patient selection criteria, or biological mechanisms of action. Findings are intended to inform future prospective research and contribute preliminary evidence for clinical discussions rather than establish definitive treatment guidelines. The single-center design and non-randomized treatment allocation limit generalizability and causal inference.

## Materials and methods

Study design

A retrospective, comparative cohort study was conducted to evaluate the clinical effectiveness of PRP versus DPT in the treatment of OSD. The study was performed at the Department of Orthopaedics, Jalappa Hospital, Sri Devaraj Urs Medical College, Kolar, Karnataka, India. This study was conducted in accordance with the ethical principles outlined in the Declaration of Helsinki and it received retrospective approval from the Institutional Ethics Committee (Approval No: SDUMCK/IEC/2024/01, dated January 15, 2024). Written informed consent was obtained from all participants and their parents or legal guardians for the retrospective analysis of medical records and publication of anonymized results. Patient confidentiality was maintained throughout the study, and all data were de-identified prior to analysis. The study was conducted in compliance with institutional guidelines and applicable regulatory requirements. Electronic medical records and follow-up reports from January 2022 to December 2024 were systematically analyzed. This study follows the Strengthening the Reporting of Observational Studies in Epidemiology (STROBE) guidelines for reporting observational studies.

Sample size calculation

Power analysis was performed using G*Power 3.1.9.7 software (Franz Faul Universitat, Kiel, Germany). Based on a pilot study of 10 patients from our institution showing a mean VAS difference of 1.5 ± 1.8 points between treatment groups, with an expected large effect size of 0.8, alpha level of 0.05, and desired power (1-β) of 0.80, the minimum required sample size was calculated as 18 patients per group. To account for potential data loss and ensure adequate power, 20 patients per group were included in the final analysis.

Patient selection

Medical records of adolescent patients aged between 10 and 18 years who received either PRP or DPT treatment for clinically and radiologically diagnosed OSD were systematically screened. From an initial cohort of 65 patients, 40 patients who met the inclusion criteria and had complete follow-up data were enrolled. Treatment allocation was non-randomized and based on multiple factors, including treatment availability at time of presentation, insurance coverage considerations, physician recommendation, and shared decision-making between patients, families, and treating physicians.

Inclusion criteria comprised age between 10 and 18 years; clinical and radiological diagnosis of OSD confirmed by anterior knee pain, swelling, and tenderness at tibial tuberosity with radiographic evidence of tibial tubercle changes; persistent symptoms for at least three months despite adequate conservative management including non-steroidal anti-inflammatory drugs (NSAIDs), physiotherapy, and activity modification for minimum eight weeks; no previous injection therapies; and complete follow-up data available for 12 weeks post-intervention.

Exclusion criteria included prior knee surgery or significant trauma; co-existing autoimmune or rheumatologic disorders; coagulopathies or hematologic conditions contraindicating PRP therapy; recent corticosteroid injection within three months prior to treatment; incomplete follow-up data or non-compliance with rehabilitation protocol; and bilateral OSD where only the more symptomatic limb was included.

Statistical analysis

All statistical analyses were performed using IBM SPSS Statistics for Windows, version 28.0 (IBM Corp., Armonk, NY). A hierarchical testing approach was employed with primary outcomes (VAS and Lysholm scores) tested using Bonferroni-adjusted significance levels (α = 0.025 for two primary outcomes, further adjusted to α = 0.0125 for four time points per outcome). Secondary outcomes were tested at α = 0.05 as exploratory analyses.

Data distribution normality was assessed using the Shapiro-Wilk test and visual inspection of Q-Q plots. Homogeneity of variance was verified using Levene's test. Continuous data were expressed as mean ± standard deviation with 95% confidence intervals. Effect sizes were calculated using Cohen's d with 95% confidence intervals, interpreted as small (0.2), medium (0.5), or large (0.8) effects.

Between-group comparisons at each time point were performed using independent-samples t-tests. Within-group changes were assessed using repeated-measures ANOVA with Greenhouse-Geisser correction when sphericity assumptions were violated. Missing data (<5% of total assessments) was handled using pairwise deletion.

## Results

Baseline characteristics

A total of 40 adolescent patients were included in the final analysis, with 20 patients in each treatment group. The groups were well matched for all baseline demographic and clinical characteristics (Table 1). The mean age was 14.6 ± 1.9 years in the PRP group and 14.3 ± 2.1 years in the DPT group, with no statistically significant difference (p = 0.62). Gender distribution was identical between groups, with 60% male patients and 40% female patients in both cohorts. The mean duration of symptoms prior to injection therapy was 4.8 ± 1.2 months in the PRP group and 5.1 ± 1.4 months in the DPT group (p = 0.47), indicating comparable chronicity of symptoms. Baseline pain and functional scores were similar between groups, with no statistically significant differences in VAS or Lysholm scores (p > 0.05), ensuring appropriate comparability for outcome analysis.

**Table 1:** Baseline Characteristics of Study Participants Data are mean ± standard deviation for continuous variables and number (percentage) for categorical variables. PRP, platelet-rich plasma; DPT, dextrose 10% prolotherapy; VAS, visual analog scale; M, male; F, female; CI, confidence interval.

Characteristic	PRP Group (n = 20)	DPT Group (n = 20)	Mean Difference (95% CI)	Test Statistic	p-Value
Mean age (years)	14.6 ± 1.9	14.3 ± 2.1	0.3 (-1.0, 1.6)	t = 0.50	0.62
Gender (M/F)	12/8	12/8	-	χ² = 0.00	1.00
Symptom duration (months)	4.8 ± 1.2	5.1 ± 1.4	-0.3 (-1.1, 0.5)	t = -0.73	0.47
Baseline VAS score	7.9 ± 0.8	7.6 ± 1.0	0.3 (-0.3, 0.9)	t = 1.09	0.28
Baseline Lysholm score	50.8 ± 6.2	52.1 ± 7.0	-1.3 (-5.4, 2.8)	t = -0.69	0.49
Sports participation					
- High-impact sports	15 (75%)	14 (70%)	-	χ² = 0.11	0.74
- Low-impact sports	5 (25%)	6 (30%)	-		
Bilateral involvement	3 (15%)	4 (20%)	-	χ² = 0.17	0.68

Pain reduction (VAS score)

Both treatment groups demonstrated significant pain reduction over the 12-week follow-up period (repeated-measures analysis of variance [ANOVA]: F = 286.4, p < 0.001). However, the PRP group showed significantly greater and more rapid pain reduction across all follow-up intervals compared to the DPT group (Table 2). The treatment-by-time interaction was statistically significant (F = 18.7, p < 0.001), indicating differential improvement patterns between groups. At week 4, the mean VAS score was 5.3 ± 0.9 in the PRP group compared to 6.1 ± 0.8 in the DPT group (p = 0.009, Cohen's d = 0.94). By week 12, PRP patients achieved a mean VAS score of 1.8 ± 0.9, significantly lower than the DPT group's score of 3.4 ± 1.1 (p < 0.001, Cohen's d = 1.60), representing a large clinically meaningful difference.

**Table 2:** Comparison of VAS Scores Over Time Data are mean ± SD with 95 % confidence intervals. Comparisons via independent‑samples t‑tests with Bonferroni correction; effect sizes are expressed as Cohen’s d. PRP, platelet-rich plasma; DPT, dextrose 10% prolotherapy; CI, confidence interval; VAS, visual analog scale.

Time Point	PRP Group (Mean ± SD)	DPT Group (Mean ± SD)	Mean Difference (95% CI)	Test Statistic	Cohen's d	p-Value
Baseline	7.9 ± 0.8	7.6 ± 1.0	0.3 (-0.3, 0.9)	t = 1.09	0.33	0.28
Week 4	5.3 ± 0.9	6.1 ± 0.8	-0.8 (-1.4, -0.2)	t = -2.78	0.94	0.009
Week 8	3.2 ± 0.9	4.6 ± 1.0	-1.4 (-2.0, -0.8)	t = -4.69	1.48	<0.001
Week 12	1.8 ± 0.9	3.4 ± 1.1	-1.6 (-2.3, -0.9)	t = -5.01	1.60	<0.001

Functional improvement (Lysholm score)

Both groups experienced significant functional improvement over the study period (repeated-measures ANOVA: F = 312.8, p < 0.001), but PRP-treated patients demonstrated significantly greater functional gains (Table 3). The treatment-by-time interaction was statistically significant (F = 15.23, p < 0.001), confirming differential improvement between groups. The PRP group improved from a baseline score of 50.8 ± 6.2 to 91.5 ± 4.8 by week 12, representing a 40.7-point improvement. In contrast, the DPT group improved from 52.1 ± 7.0 to 82.4 ± 5.6, representing a 30.3-point improvement. The between-group difference at week 12 was statistically significant (p < 0.001) with a large effect size (Cohen's d = 1.71).

**Table 3:** Lysholm Knee Score Over Time Data are mean ± SD with 95 % confidence intervals. Between‑group comparisons via independent‑samples t‑tests with Bonferroni correction; effect sizes are expressed as Cohen’s d. PRP, platelet-rich plasma; DPT, dextrose 10% prolotherapy; CI, confidence interval.

Time Point	PRP Group (Mean ± SD)	DPT Group (Mean ± SD)	Mean Difference (95% CI)	Test Statistic	Cohen's d	p-Value
Baseline	50.8 ± 6.2	52.1 ± 7.0	-1.3 (-5.4, 2.8)	t = -0.69	0.20	0.49
Week 4	68.4 ± 5.9	63.1 ± 6.4	5.3 (1.4, 9.2)	t = 2.75	0.86	0.009
Week 8	79.8 ± 5.6	73.9 ± 6.1	5.9 (2.2, 9.6)	t = 3.25	1.02	0.003
Week 12	91.5 ± 4.8	82.4 ± 5.6	9.1 (5.9, 12.3)	t = 5.47	1.71	<0.001

Return to sports

PRP patients demonstrated significantly faster return to unrestricted sports participation compared to DPT patients (Table 4). The mean time to return to sports was 6.1 ± 1.2 weeks in the PRP group versus 8.5 ± 1.3 weeks in the DPT group. This 2.4-week difference was statistically significant (t = 5.92, p < 0.001) with a very large effect size (Cohen's d = 1.94), indicating both statistical and clinical significance of the finding.

**Table 4:** Time to Return to Sports Data are mean ± SD, 95% confidence intervals, and median (interquartile range). Group differences are assessed by independent‑samples t‑test; effect size is expressed as Cohen’s d. IQR, interquartile range; CI, confidence interval; PRP, platelet-rich plasma; DPT, dextrose 10% prolotherapy.

Group	Mean ± SD (Weeks)	95% CI	Median (IQR)	Test Statistic	Cohen's d	p-Value
PRP group	6.1 ± 1.2	5.6-6.6	6.0 (5.0-7.0)	t = 5.92	1.94	<0.001
DPT group	8.5 ± 1.3	7.9-9.1	8.5 (7.0-9.0)			

Patient satisfaction

Patient satisfaction assessment at week 12 revealed significantly higher satisfaction rates in the PRP group compared to the DPT group (Table 5). Eighteen out of 20 patients (90%) in the PRP group reported being satisfied with their treatment outcome (Likert scores 4-5), compared to 13 out of 20 patients (65%) in the DPT group. This difference was statistically significant (χ² = 4.27, p = 0.039). The odds of treatment satisfaction were 5.54 times higher in the PRP group compared to the DPT group (OR = 5.54; 95% CI: 1.01-30.2). The number needed to treat (NNT) was calculated as 4, indicating that for every four patients treated with PRP instead of DPT, one additional patient would achieve treatment satisfaction.

**Table 5:** Satisfaction Level at Week 12 Data are n (%). Between‑group comparison is done using the chi‑square test; OR with 95% CI and NNT are reported. OR, odds ratio; CI, confidence interval; PRP, platelet-rich plasma; DPT, dextrose 10% prolotherapy; NNT, number needed to treat.

Satisfaction Level	PRP Group n (%)	DPT Group n (%)	OR (95% CI)	Test Statistic	NNT	p-Value
Satisfied (Likert 4-5)	18 (90%)	13 (65%)	5.54 (1.01-30.2)	χ² = 4.27	4	0.039
Not Satisfied (Likert ≤3)	2 (10%)	7 (35%)				

Adverse events

No serious adverse events were reported in either treatment group during the 12-week follow-up period. Minor side effects included temporary injection-site pain lasting 24-48 hours (PRP: 3 patients [15%], DPT: 2 patients [10%]), mild localized swelling resolving within 48 hours (PRP: 2 patients [10%], DPT: 1 patient [5%]), and transient bruising at injection sites (PRP: 1 patient [5%], DPT: 0 patients). All reported adverse events were mild in severity and resolved spontaneously without requiring medical intervention or affecting treatment outcomes.

Correlation analysis

Strong positive correlation was observed between VAS score reduction and Lysholm score improvement across all study participants (Pearson's r = 0.78, p < 0.001), indicating that greater pain reduction was consistently associated with superior functional improvement regardless of treatment modality.

## Discussion

This retrospective comparative study provides preliminary evidence regarding the clinical utility of regenerative therapies in adolescents with refractory OSD. The results suggest that PRP injections may offer advantages over DPT across multiple clinically relevant domains, including pain reduction, functional improvement, return-to-sports timeline, and patient satisfaction. However, the study design limitations require cautious interpretation of these findings.

The observed differences in pain relief with PRP therapy, as evidenced by large effect sizes (Cohen's d >1.4) from week 8 onwards, may reflect the biological mechanisms mediated by concentrated growth factors present in platelet preparations [19,20]. However, this study did not measure biological markers to confirm mechanistic hypotheses. The high concentrations of platelet-derived growth factor, vascular endothelial growth factor, and transforming growth factor-beta in PRP theoretically create an optimal microenvironment for tissue healing [21], though the clinical benefits observed could also result from placebo effects, natural disease progression, or unmeasured confounding factors inherent in the non-randomized design.

The functional improvements observed in both groups, as measured by Lysholm scores, suggest the regenerative potential of both therapies. The PRP group achieved excellent functional scores (>90) by week 12, while the DPT group reached good functional levels (82.4). Whether this difference translates to meaningful long-term outcomes remains unknown, given the limited follow-up period. The strong correlation between pain reduction and functional improvement (r = 0.78, 95% CI: 0.62-0.88) suggests that effective symptom management may translate into functional gains, though this relationship requires validation in prospective studies [22].

The faster return to sports in the PRP group (6.1 vs 8.5 weeks) may represent a clinically meaningful advantage, though this outcome is susceptible to bias in retrospective designs where return-to-sport decisions may be influenced by patient and provider expectations. The 2.4-week difference, while statistically significant, requires validation in blinded, controlled studies to confirm clinical relevance [23].

Comparison with existing literature

Direct comparative studies of PRP versus DPT in pediatric OSD are lacking, limiting our ability to contextualize these findings. While systematic reviews demonstrate moderate evidence supporting PRP efficacy in adult tendinopathies [24], direct extrapolation to pediatric populations may not be appropriate, given differences in healing responses, pain perception, and treatment compliance between age groups. Recent pediatric studies on regenerative therapies remain limited, representing a significant knowledge gap in adolescent sports medicine [25].

The effectiveness of DPT observed in our study aligns with previous reports in musculoskeletal conditions, though most evidence comes from adult populations [26]. The delayed response pattern observed with DPT may reflect its indirect mechanism through induced proliferation rather than direct growth factor-mediated healing, though this mechanistic explanation requires validation through prospective studies with biological outcome measures.

Clinical implications and treatment selection

These preliminary findings suggest that PRP may offer advantages over DPT for refractory OSD, though larger randomized trials are needed before establishing treatment preferences [27]. Treatment selection should remain individualized based on multiple factors, including symptom severity, athletic demands, economic considerations, patient preferences, and institutional expertise.

DPT remains a potentially valuable treatment option, particularly in resource-constrained settings or for patients with less severe symptoms. Its lower cost and simpler preparation may provide accessibility advantages, though the multiple injection protocol required for DPT may present challenges for some patients and families.

Study limitations

Several important limitations must be acknowledged when interpreting these findings. The retrospective design introduces inherent selection bias and limits the ability to control for unmeasured confounding variables that may have influenced treatment allocation and outcomes [28]. The non-randomized treatment assignment based on availability and preference may have created systematic differences between groups despite similar baseline characteristics.

The relatively small sample size (n = 40) limits statistical power for subgroup analyses and external validity. The 12-week follow-up period is insufficient to evaluate long-term outcomes, recurrence rates, or potential delayed complications. The single-center design may limit generalizability across different healthcare systems and patient populations.

Assessment bias may have been introduced through reliance on subjective outcome measures, though validated scales were employed. The study population was limited to patients with refractory OSD, potentially representing a more severe subset and limiting generalizability to patients with milder symptoms. Long-term safety data and potential impacts on growth plate development remain unknown and require prospective longitudinal investigation.

## Conclusions

This retrospective comparative study provides preliminary evidence suggesting potential advantages of PRP therapy over DPT in managing adolescents with refractory OSD. PRP demonstrated statistically significant and clinically meaningful improvements in pain reduction, functional recovery, return to sports participation, and patient satisfaction compared to DPT over a 12-week follow-up period. The large effect sizes observed (Cohen's d >1.4) indicate substantial clinical benefits, with PRP patients achieving excellent functional scores and returning to sports approximately 2.4 weeks earlier than DPT patients.

However, several important limitations must be acknowledged. The non-randomized retrospective design, small sample size (n = 40), single-center setting, and short follow-up period significantly limit the strength of these conclusions and the generalizability of findings. Treatment allocation based on availability and preference may have introduced systematic bias despite similar baseline characteristics between groups. Long-term safety and efficacy outcomes remain unknown.

Both interventions demonstrated favorable short-term safety profiles, supporting their potential as minimally invasive alternatives to surgical management in appropriately selected patients. While these findings contribute valuable preliminary data to the limited literature on regenerative therapies in pediatric OSD, definitive treatment recommendations require confirmation through larger, multicenter, randomized controlled trials with extended follow-up periods. Until such evidence becomes available, treatment decisions should remain individualized based on patient-specific factors, family preferences, and shared clinical decision-making.
